# Digital Panoramic Radiographs for Age Prediction Utilizing the Tooth Coronal Index of First Mandibular Bicuspids Among the South Indian Population

**DOI:** 10.7759/cureus.45870

**Published:** 2023-09-24

**Authors:** Hooriyah Laiq Ahmed Khan, Karthikeyan Murthykumar, Saravanan Sekaran, Dhanraj Ganapathy

**Affiliations:** 1 Prosthodontics, Saveetha Dental College and Hospitals, Saveetha Institute of Medical and Technical Sciences, Saveetha University, Chennai, IND; 2 Periodontics, Saveetha Dental College and Hospitals, Saveetha Institute of Medical and Technical Sciences, Saveetha University, Chennai, IND

**Keywords:** digital orthopantomogram, bicuspids, teeth, forensics, health care, age estimation

## Abstract

Introduction

Age estimation holds significant importance within the realm of forensic science, serving as a crucial tool for various purposes such as validating birth certificates, aiding immigration processes, and determining eligibility for retirement benefits. Additionally, age estimation carries significant implications in situations involving human trafficking, offering insights into matters such as legal culpability, adult classification, and marriage age assessment.

Aim

The purpose of this research was to assess the precision of the Tooth Coronal Index (TCI) in the estimation of age, a key component of forensic odontology.

Materials and methods

The research employed a retrospective approach, analyzing 700 digital panoramic radiographs of the mandibular first bicuspids. The study population was categorized into five age groups viz. 20-30, 31-40, 41-50, 51-60, and above 61 years respectively. Statistical methods were applied to investigate the relation between TCI and age. Additionally, one-way ANOVA was utilized to compare the groups.

Results

Findings revealed that males aged between 20-30 years exhibited underestimation, while males above 60 years displayed overestimation. Among females, the smallest disparity between existent and calculated age was observed in the 31-40 age group. Notably, ANOVA analysis for females indicated highly significant differences between the calculated and actual ages across all age segments (P<0.01). Regarding the mean TCI, inter-group comparisons showed statistically insignificant differences in males, while in females, the distinctions were statistically extremely noteworthy (P<0.01).

Conclusion

The utilization of TCI for age estimation based on mandibular first bicuspids is recommended as a convenient, non-invasive, and time-efficient approach.

## Introduction

Age estimation is a pivotal focus within the realm of forensic science, offering critical insights for applications ranging from birth certificates and immigration to retirement assistance. It forms a fundamental aspect of forensic anthropology to construct a biological profile for individuals. The significance of age estimation also extends to addressing issues like human trafficking, aiding in the determination of criminal responsibility, legal adulthood, and marital age. Numerous techniques exist for age estimation, encompassing chronological age, skeletal age, and dental age. Among these, the Teeth Coronal Index (TCI) stands as a method employed in forensic odontology to estimate age. This technique involves the examination of secondary dentin deposition on the roof of the pulpal chamber within a tooth [1].

As an individual grows older, an increased deposition of secondary dentin occurs, which can be quantified and contrasted with established reference data to approximate their age. This technique is frequently employed to deduce the age of deceased individuals or in situations where age determination is reliant on dental remnants. For sub-adult individuals, age estimation postmortem involves evaluating the fusion of secondary ossification centers and the progress as well as the emergence of teeth. In contrast, gauging the age of adults is a more intricate process, underscoring the significance of forensic odontology in this endeavor [2].

Different types of medical imaging techniques like radiography, computed tomograms, and magnetic resonance imaging are used as skeletal indicators of age. These indicators encompass the fusion of the distal part of the clavicle, sutures in the skull, the junction of the pelvic bones, and the conversion of cartilage in the ribs into bone. These indicators aid in estimating the age of living individuals [2]. Among these indicators, teeth hold the highest level of reliability due to their resistance to taphonomic processes and slow disintegration. Furthermore, dental pulp can serve as an age indicator as it diminishes in size with age owing to continuous dentin deposition [3]. Ikeda et al. introduced the TCI as a straightforward, non-invasive technique for estimating age through radiological assessment of teeth, circumventing the need for tooth extraction [4]. Subsequently, Drusini evaluated the accuracy of TCI on a cohort of 433 individuals, validating its efficacy as a dependable tool for age determination [5]. Numerous subsequent studies have investigated the efficacy and accuracy of the TCI across diverse populations in various geographic regions [6]. Karkhanis et al. ascertained the utility of TCI for forensic purposes in an Australian population. However, equations tailored for precise age estimation based on measurements of secondary dentin deposition, initially devised for the Western population, yielded unsatisfactory errors when extrapolated in broader Indian population [7]. Koranne et al. established TCI regression equations were applicable for age estimation among individuals aged 20 to 60 years, demonstrating no gender-based variations in TCI [8]. Therefore, the objective of this research aimed to estimate and predict age through measurement of mandibular bicuspid on panoramic radiographs, while also evaluating the dependability of TCI in estimating age within a south Indian population.

## Materials and methods

**Figure 1:**
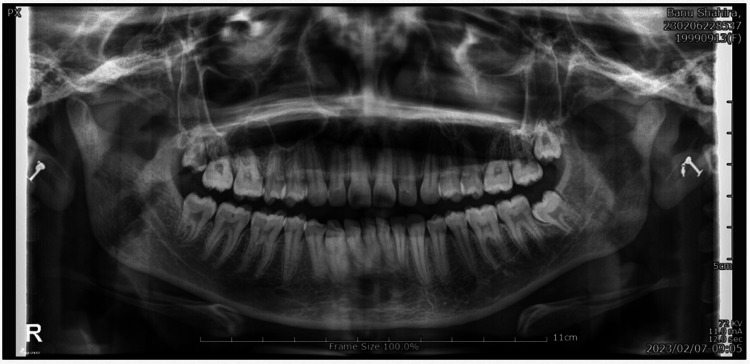
Digital OPG used for assessment OPG: Orthopantomograph

The study was conducted in the Department of Prosthodontics at Saveetha Dental College and Hospitals, Chennai, India. The Institutional Ethical Committee provided approval for the implementation of this retrospective study. The research involved the assessment of Digital Orthopantomographs (OPG) from adult patients who visited a dental college seeking diagnostic assistance for their primary complaints between the years 2022 and 2023. The panoramic radiographs were taken using a KODAK 9000C 3D Unit, operating at 70-80 kVp and 10 mA, with an image capture time of 13.8 seconds. The inclusive sample consisted of the Digital OPG images of 700 patients. The inclusion criteria dictated an age range spanning from 20 to 70 years, subsequently segregated into five distinct age groups viz. 20-30, 31-40, 41-50, 51-60, and above 61 years respectively. For analysis, the permanent mandibular first bicuspid (either left or right) was selected from the panoramic radiographs. Conversely, certain exclusion criteria were established, encompassing teeth displaying pathologies like caries, periodontitis, or periapical lesions, as well as those that underwent restorative, endodontic, or prosthetic treatment. In this study, if a mandibular first bicuspid exhibited a shape abnormality, such as an unusually pointed or irregular crown shape, it would be considered an exclusion criterion. Additionally, teeth exhibiting substantial rotation or enamel overlap, along with developmental abnormalities pertaining to size, shape, or structure, were also excluded. The TCI, as proposed by Ikeda et al., was implemented for age estimation. The TCI method relies on two linear measurements. A straight line was sketched between the cemento-enamel junctions on the mesial and distal aspects of all teeth that were measured, serving as the basis for anatomical crown evaluation. The data obtained from the digital panoramic radiograph for the mandibular right/left first bicuspid included the following measurements (Figure 1):

i) Crown height (CH): assessed vertically from the cervical margin to the apex of the tallest cusp. ii) Coronal pulp cavity height (CPCH): evaluated vertically from the cervical line to the tip of the highest pulp horn.

iii) TCI calculation: TCI = (CPCH × 100) / CH.

In panoramic radiographs, blurring was evident in the anterior teeth region, leading to the acquisition of projections from just one angle. Consequently, the selection of bicuspids was favored. Mandibular teeth were prioritized due to their superior visibility compared to maxillary teeth on panoramic radiographs. This approach aligned with previous studies. The first bicuspids were chosen for their attributes as single-rooted teeth with substantial pulp space, making them convenient for measurements and morphometric analysis. The utilization of bicuspids was informed by their demonstrated correlation with age. While the literature on the exclusive use of mandibular first bicuspids for age estimation is scarce, this study aimed to evaluate their reliability for this purpose within the adult population. Three proficient observers, each possessing comparable expertise in orofacial radiology, conducted the measurements. The combined findings from these observers were collected and structured for subsequent statistical assessment using SPSS (IBM Corp., Armonk, NY, USA). The final CPCH and CH values were derived by averaging the measurements provided by the three observers. TCI was computed using the formula TCI = (CPCH × 100) / CH.

## Results

The complete dataset encompassed panoramic radiographs from 700 adult individuals, including 388 males and 312 females The subjects' ages spanned from 20 to 70 years and were divided into five separate segments and the average age of the participants in the study was calculated to be 31.56±10.924 years. The dataset was subjected to determine normality using the Shapiro-Wilk test of normality and it was observed the data had a normal distribution (p < 0.05) (Table 1). The distribution of the mean TCI for different age groups is shown in Table 1.

**Table 1:** Mean TCI for different age groups. N: Number of patients enrolled in the study, TCI: Tooth Coronal Index, SD: Standard Deviation, SE: Standard Error

Age(y)	N	Mean TCI	SD	SE	Min	Max
20-30	399	35.45	4.89	0.24	0	48
31-40	163	37.45	6.37	0.5	21	74
41-50	78	35.58	7.21	0.8	21	77
51-60	52	36.14	6.46	0.91	20	50
>61	8	36.38	7.19	2.63	24	48
Total	700	36.20	6.42	0.21	0	77

The association between age and TCI across distinct age groups was assessed through an ANOVA test. The outcomes indicated a highly significant statistical difference in the inter-group comparison of age and mean TCI (P<0.01). Further ANOVA analysis demonstrated that among males, the inter-group comparison of mean TCI did not yield statistically significant differences (P>0.05). However, within the female cohort, a pronounced and statistically highly significant disparity was observed with mean TCI (P<0.01) (Table 2).

**Table 2:** ANOVA for group comparison

	TOOTH CORONAL INDEX				
		Sum of squares	Degrees of freedom (df)	F-value	P-value
Male	Between groups	158.68	4	1.15	0.324
	Within groups	13096.7	384	0	0
	Total	13255.42	388	0	0
Female	Between groups	463.11	4	0	0
	Within groups	9263.55	306	3.45	0.005
	Total	9726	310	0	0

The assessment of the average deviations from actual age across different age groups for both males and females unveiled distinct patterns. Among male participants, age underestimation occurred in group 1, while age overestimation was observed in group 5. Notably, the largest average deviation was noted in age group 5 for males, indicating potential inaccuracy of the formula in older age categories. ANOVA analysis for inter-age comparison in males demonstrated no statistically significant differences in the deviations from actual age across all age groups (P>0.05).

In the female cohort, it was evident that the age estimation formula yielded the smallest deviation from actual age in group 2, with the most substantial deviation occurring in age group 5. Subsequently, inter-age comparison for females unveiled a highly significant statistical difference in the deviations from actual age across all age groups (P<0.01) (Tables 3, 4).

**Table 3:** Evaluation of variance in mean disparities between calculated and actual ages across various age categories within the male and female groups. TCI: Tooth Coronal Index, SD: Standard Deviation, SE: Standard Error

					95%CI for mean		Difference from actual age	
Gender	Age(y)	Mean TCI	SD	SE	Min	Max	Min	Max
Males	20-30	-2.61	2.35	1.05	-5.54	0.3	-4.48	7.55
	31-40	2.65	3.24	1.45	-1.37	6.68	-4.48	7.55
	41-50	1	4.45	1.99	-4.52	6.53	7.19	17.2
	51-60	3.06	2.61	1.16	-0.18	6.3	0.74	4.76
	>61	3.3	3.85	1.72	-1.47	8.08	-17.85	-11.84
	Total	1.48	3.82	0.76	-0.09	3.06	-25.44	-19.2
Females	20-30	14.501	4.19	1.87	9.29	19.71	12.45	30.26
	31-40	2.75	1.87	0.83	0.42	5.07	0.74	4.76
	41-50	-15.32	2.26	1.01	-18.13	-12.51	-17.85	-11.84
	51-60	-22.7	2.27	1.01	-25.53	-19.87	-25.44	-19.2
	>61	21.43	7.38	3.3	12.26	30.6	12.45	30.26
	Total	0.13	17.65	3.53	-7.15	7.418	-25.44	30.26

**Table 4:** Comparison of TCI (mm) between different age groups using ANOVA for both males and females TCI: Teeth Coronal Index

	Difference from Actual Age				
		Sum of squares	Degrees of freedom (df)	F- value	Significance (P-value)
Male	Between groups	121.21	4	2.61	0.065
	Within groups	230.29	20	0	0
	Total	351.51	24	0	0
Female	Between groups	7137.31	4	0	0
	Within groups	343.84	23	0	<0.01
	Total	7481.15	24	0	0

## Discussion

In 1985, Ikeda et al. pioneered the creation of the Tooth Crown Index (TCI). This metric involved gauging the length of the crown and coronal pulp in extracted human teeth and subsequently evaluating radiographic prints. The correlation coefficients for female molars and bicuspids were reported as -0.73 and -0.89, respectively [4]. Furthermore, using gender-specific formulas to estimate age did not yield statistically significant differences.

The findings of this study aligned with prior research carried out by Badar et al. on a group from Pakistan. Their study unveiled an average correlation coefficient (r) of -0.27 between chronological age and TCI, suggesting a remarkably weak association between age and TCI [6]. Similarly, the findings aligned with the work of Karkhanis et al. in the Australian subpopulation. Their employment of multiple regression analysis identified the mandibular right first bicuspid as consistently displaying the highest correlation coefficient (r = -0.262) for the combined sample [7]. Likewise, similar observation focusing on Indian populations, which assessed the effectiveness of diverse age estimation formulas, demonstrated results akin to those observed in our study [9].

Jain et al. identified a negative correlation between chronological age and Tooth Crown Index (TCI) for both the mandibular first molar and the second bicuspid [10]. Hatice et al., in their examination of a Turkish population, found a correlation of -0.230 between TCI and age for the mandibular first bicuspid [11]. Notably, the present study was carried out in a bustling southern metropolis, resulting in a diverse and heterogeneous population. The heightened precision in the investigation conducted by Drusini et al. might be due to the homogeneous nature of their study group. Interestingly, our findings diverged from those of Drusini with correlations ranging from -0.73 to -0.89, as well as from the results of Igbigbi and Nyirenda's research in Malawi with correlations of -0.650 to -0.799, and observations by Talabani et al. with an r^2 value of 0.49 [12, 13]. The variation in correlation coefficients could be ascribed to the differences and inconsistencies in dental development among individuals in the same population and across diverse regional multiethnic groups. The quality and extent of secondary dentin deposition are influenced by factors such as race, ethnicity, background, dietary habits, and lifestyle [14-19]. The impact of secondary factors on the TCI in terms of dimensions could arise from either traditional or digital radiographic methods. In the context of digital radiography, external and ambient lighting, specific attributes of computer monitors, as well as computer hardware and software characteristics, might influence how observers select reference points for measurements, consequently affecting TCI outcomes. Applying the equations obtained from the regression analysis that linked TCI and age to a cohort of 50 participants (consisting of 25 males and 25 females), it was observed that 18 of the male participants and five of the female participants exhibited a variance of under 5 years from their real age. This indicated that TCI appeared to offer greater accuracy for males when compared with females [20].

Limitations

However, it's important to acknowledge this study’s limitation, namely that the radiographs utilized in the research were representative of a heterogeneous regional-based Southern Indian population and might not fully represent the wider national population. Additionally, with the emergence of Cone Beam Computed Tomography (CBCT), many researchers might prefer CBCT over orthopantomograms (OPG) for the estimation of age. Nevertheless, this study endorsed the application of OPGs since they were selected based on the primary concerns of patients during their hospital visits and provided assurance of no additional exposure to ionizing radiation. To advance the field of dental age estimation formulas, future studies are encouraged to include larger sample sizes and consider teeth other than mandibular first bicuspids. Furthermore, the use of multiple regression analysis would be beneficial for such investigations.

## Conclusions

The findings of this research hold validity within the scope of the relatively small sample size and indicate that TCI exhibited better accuracy for males compared to females. While TCI demonstrated precision in estimating ages within the range of 31 to 40 years, its accuracy was diminished for individuals aged 20 to 30 and those beyond 61 years. To enhance the accuracy and reliability of results, and to develop a universally applicable formula, it is recommended that similar studies be undertaken on a larger and more comprehensive sample. Such studies should encompass a broader range of ages, ethnicities, and genders to minimize standard errors and attain maximum reproducibility.
